# What is the optimal time-response window for the use of photobiomodulation therapy combined with static magnetic field (PBMT-sMF) for the improvement of exercise performance and recovery, and for how long the effects last? A randomized, triple-blinded, placebo-controlled trial

**DOI:** 10.1186/s13102-020-00214-8

**Published:** 2020-10-19

**Authors:** Ernesto Cesar Pinto Leal-Junior, Marcelo Ferreira Duarte de Oliveira, Jon Joensen, Martin Bjørn Stausholm, Jan Magnus Bjordal, Shaiane Silva Tomazoni

**Affiliations:** 1 Laboratory of Phototherapy and Innovative Technologies in Health (LaPIT), Post-graduate Program in Rehabilitation Sciences, Nove de Julho University, Rua Vergueiro, 235/249, São Paulo, SP 01504-001 Brazil; 2 Physiotherapy Research Group, Department of Global Public Health and Primary Care, University of Bergen, Bergen, Norway; 3 ELJ Consultancy - Scientific Consultants, São Paulo, Brazil

**Keywords:** Phototherapy, Low-level laser therapy, Light emitting diodes, Performance, Post-exercise recovery

## Abstract

**Background:**

The optimal time-response window for photobiomodulation therapy (PBMT) using low-level laser therapy (LLLT) and/or light emitting diodes therapy (LEDT) combined with static magnetic fields (sMF) before physical activity still was not fully investigated. The aim of the present study was to investigate the better of four time-response windows for PBMT combined with sMF (PBMT-sMF) use before exercise in humans.

**Methods:**

A prospectively registered, randomized, triple-blinded (volunteers, therapists and assessors) placebo-controlled trial was carried out. Sixty healthy untrained male subjects were randomly allocated to six experimental groups (*n* = 10 per group): PBMT-sMF 5 mins, PBMT-sMF 3 h, PBMT-sMF 6 h, PBMT-sMF 1-day, placebo, and control. The control group performed all procedures, however did not receive any kind of intervention. PBMT-sMF active or PBMT-sMF placebo was applied precisely in different time points after baseline MVC test to ensure that both MVC tests and eccentric exercise protocol would occur at the same hour of the day in all groups. Then, after five minutes, 3 h, 6 h or 1-day (24 h) of PBMT-sMF treatment (active or placebo) the eccentric exercise protocol was performed. The primary outcome was peak torque obtained from maximum voluntary contraction (MVC). The secondary outcomes were creatine kinase (CK), and delayed onset muscle soreness (DOMS). The primary and secondary outcomes were measured at baseline, immediately after, 1 h, 24 h and 48 h after the eccentric exercise protocol.

**Results:**

Sixty patients were randomized and analyzed to each sequence. The outcomes in absolute values show that all active PBMT-sMF groups increased (*p* < 0.05) MVC from immediately after to 1 h after eccentric exercise, and decreased (p < 0.05) CK activity at all time points. However, PBMT-sMF 5 mins, 3 h and 6 h groups showed better results in MVC and CK analysis from 24 h to 48 h, and also to DOMS (*p* < 0.05) at all time points. Participants did not report any adverse events.

**Conclusions:**

PBMT-sMF can be used from 5 min to 6 h before exercise, and the effects can last up to 54 h after treatment. However, the effects start to decrease when a 1-day (24 h) time-response window is used.

**Trial registration:**

NCT03420391. Registered 05 February 2018.

## Background

Photobiomodulation therapy (PBMT), also known as phototherapy, is a light therapy that uses non-ionizing light sources, such as lasers (light amplification by stimulated emission of radiation), light-emitting diodes (LEDs), and broadband light, from the visible to the infrared spectrum [1]. PBMT is a nonthermal process where light interacts with chromophores leading to photophysical and photochemical events in different tissues [1]. This process leads to pain relief, modulation of inflammatory process in both chronic and acute phases, wound healing [1], exercise performance enhancement and faster post-exercise recovery (known as ergogenic effects) [2]. The mechanism of action of PBMT is related to increased mitochondrial function trough modulation of cytochrome c oxidase [3], increased microcirculation [4] and decreased axonal flow [5].

Since the year of 2008 the effects of PBMT to the improvement of exercise performance and for the exercise recovery through protection of skeletal muscles against damage has been investigated in humans [6]. The first clinical trial in this field showed that PBMT before an exercise session could enhance the performance of high-level volleyball athletes (number of repetitions), decrease the delayed onset muscle fatigue (time until exhaustion), and prevent the expected increase of blood lactate levels [6]. After this pioneering clinical trial [6], PBMT used alone or in combination to static magnetic fields (sMF) have shown positive results in different kinds of exercises and protocols such as repeated contractions [6, 7], isometric contractions [8], progressive-intensity running [9–11], cycling [12, 13], eccentric contractions [14–17], strength training [18, 19], endurance training in treadmill [20] and even in field tests [21]. Regarding exercise recovery, both PBMT and PBMT-sMF have shown better effects than other agents classically used for this aim, such as cryotherapy for instance [22–24].

Static magnetic fields (sMF) are generated wherever direct currents are used [25]. sMF can interact with biological systems [25, 26] moving magnetic materials and charges found in tissues (ions and proteins for instance) through different physical mechanisms [27], such as electrodynamic interactions with ionic conduction currents, magnetomechanical interactions, and effects on electronic spin states of reaction intermediates [for more details read [25]]. These interactions result in many physiological effects, and some of them are helpful for ergogenic aims, such as increased production of adenosine triphosphate (ATP) and increased cell metabolism [28]. Over the past years, sMF has been used combined with PBMT since there is a synergistic action between them, generating a greater transfer of electrons [28]. There is evidence that PBMT combined with sMF (PBMT-sMF) is able to promote ergogenic effects in a similar way to isolated PBMT [19–21, 29–31]. Currently there is an increasing body of evidence for the use of both isolated PBMT and PBMT-sMF [32, 33] with several aspects regarding the application of these therapies being elucidated, such as: optimal doses [12, 13, 15, 16], optimal power output [17], and even comparisons between different devices commercially available [34]. However, the time-response window is still a matter of debate.

The time-response window (time interval between treatment/irradiation and the physical activity) is paramount to achieve optimal outcomes using PBMT and PBMT-sMF. An inadequate time-response window can lead to lack of biological response considering that the PBMT or PBMT-sMF effects were not elicited yet (due a short interval between the treatment/irradiation and the physical activity), or the effects are already gone (due a long interval between the treatment/irradiation and the physical activity). However, the time-response window for the use of PBMT either isolated or combined with sMF (PBMT-sMF) before physical activity still was not fully investigated in clinical scenario, and there is much speculation and overstatements regarding this matter. In fact, the only other randomized controlled trial investigating the time-response window was limited by focusing on the immediate effects of two time-response windows of PBMT on muscle fatigue [35]. Additional research is needed to understand more time window responses and the multi-day effects on delayed onset muscle soreness and biochemical markers. These aspects could expand the body of knowledge in this field and also provide a better understanding regarding another important aspect: how long the effects last after performing the exercise.

Therefore, in order to provide a better and comprehensive understanding concerning this aspect, the aim of this study was to investigate the better of four time-response windows for the use of PBMT-sMF when applied before exercise in humans, and to analyze the effects up to 48 h after performing the exercise.

## Methods

### Study design and ethics

We performed a prospectively registered (NCT03420391), randomized, triple-blinded (volunteers, therapists and assessors), placebo-controlled trial. We submitted the present study to the Regionale Komiteer for Medisinsk og Helsefaglig Forskningsetikk and it was approved under number 2019/595 REK Nord. We informed all eligible volunteers of the objective and procedures of the study, and all participants signed the written informed consent before enrollment in the study. This study adheres to CONSORT guidelines [36].

### Volunteers and recruitment

We recruited sixty healthy untrained male subjects to participate in the study. The choice to recruit only male volunteers is to allow direct comparison with previous studies carried out by our research group using a similar technology [15, 23].

### Eligibility criteria

Inclusion criteria: male volunteers between the ages of 18 and 35 were included in the study if they perform up to one exercise session per week.

Exclusion criteria: volunteers were excluded if they presented with any musculoskeletal injury to hips or knees within the previous 2 months, if they were currently using pharmacological agents or nutritional supplements regularly, if a musculoskeletal injury during the study occurred, or if they reported the use of either alcohol or tobacco.

### Randomization and blinding

We carried out the randomization using the website *random.org*. The researcher responsible for the randomization was also responsible for programming the PBMT-sMF device into active therapy or placebo. Moreover, this same researcher coded the treatments according to the randomization table without disclosing to anyone involved in the study. Since the sounds and information displayed in the device’s screen were identical, it was not possible to distinguish the mode used in the device (active or placebo), which allowed to keep the blindness of the study. The allocation was concealed by using consecutively numbered, sealed, and opaque envelopes. We randomly allocated the volunteers into six experimental groups (*n* = 10 per group): 1) PBMT-sMF 5 mins, 2) PBMT-sMF 3 h, 3) PBMT-sMF 6 h, 4) PBMT-sMF 1-day, 5) placebo, and 6) control. The active PBMT-sMF programs and placebo PBMT-sMF treatments/programs were performed using the same device. For the placebo treatments, the infrared laser diodes, the infrared LED diodes, and the magnetic field were deactivated (turned off), and the power of the red LED diodes were decreased to 0.5 mW (mean power for each diode) in order to keep the visual aspect of red light, but not to deliver an effective therapeutic or considerable dose (1.38 J to the thigh) according the current available evidence [2, 33]. A similar placebo method has been employed in several studies performed previously using the same PBMT-sMF technology [10, 15, 19–21, 23, 29–31, 34]. To further maintain blinding, participants wore opaque goggles to prevent them from seeing whether a light was being irradiated. Therefore, therapist, assessors and volunteers were blinded to which programs provided active or placebo PBMT-sMF.

### Interventions

#### Photobiomodulation therapy (PBMT) combined with static magnetic fields (sMF) (PBMT-sMF) – active

**Fig. 1:**
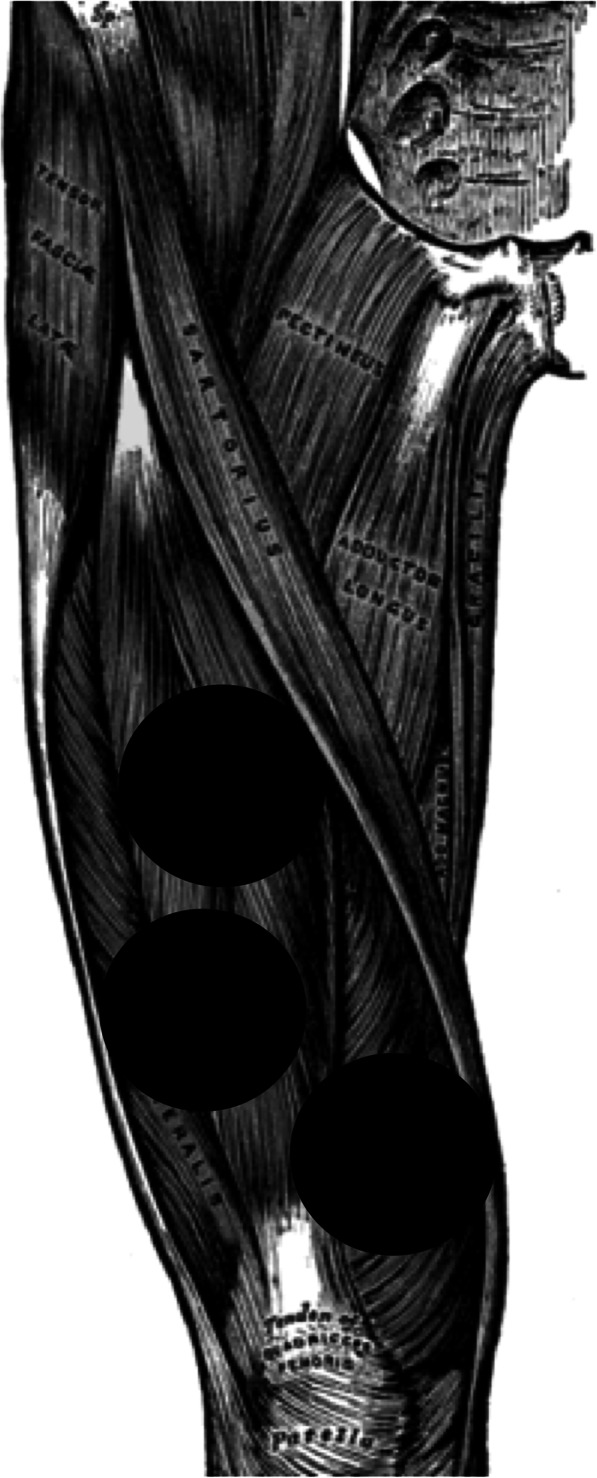
Sites of PBMT-sMF irradiation on quadriceps

We performed the PBMT-sMF treatments employing a cordless, portable MR5 Activ Pro LaserShower™ device (manufactured by Multi Radiance Medical™, Solon - OH, USA) to 3 sites of quadriceps femoris in a standardized sequence: 1 - one site medially (vastus medialis); 2 - one site laterally (vastus lateralis); and 3 - one site centrally (rectus femoris and vastus intermedius). Additionally, these three sites were located at the bottom (medially - vastus medialis), at the middle (laterally - vastus lateralis) and at the top (centrally - rectus femoris and vastus intermedius) of the distal half of volunteers’ thighs in direct contact with the skin (Fig. 1).

We chose the PBMT-sMF parameters based on parameters previously tested and optimized [10, 15, 19–21, 23] using this same technology. In some instances, the outcomes of PBMT-sMF may be impacted by skin pigmentation due to rapidly increasing surface temperature. However, the kind of PBMT-sMF technology used in this study has demonstrated previously a lack of damaging thermal effects that is independent of amount of skin pigmentation [37]. Therefore, we did not excluded volunteers due to skin color and no adjustment in PBMT-sMF parameters was needed. Moreover, we also checked the optical power before irradiation in each volunteer using a Thorlabs thermal power meter (Model S322C, Thorlabs®, Newton - NJ, USA). The full description of parameters is provided in Table 1.

**Table 1:** PBMT-sMF parameters

Number of lasers	4
Wavelength (nm)	905
Frequency (Hz)	250
Peak power (W) - each	50
Average mean optical output (mW) - each	1.25
Power density (mW/cm^2^) - each	3.91
Energy density (J/ cm^2^) - each	0.45
Dose (J) - each	0.144
Spot size of laser (cm^2^) - each	0.32
Number of red LEDs	8
Wavelength of red LEDs (nm)	633
Frequency (Hz)	2
Average optical output (mW) - each	25
Power density (mW/cm^2^) - each	29.41
Energy density (J/ cm^2^) - each	3.38
Dose (J) - each	2.875
Spot size of red LED (cm^2^) - each	0.85
Number of infrared LEDs	8
Wavelength of infrared LEDs (nm)	850
Frequency (Hz)	250
Average optical output (mW) - each	40
Power density (mW/cm^2^) - each	71.23
Energy density (J/ cm^2^) - each	8.214
Dose (J) - each	4.600
Spot size of infrared LED (cm^2^) - each	0.56
Magnetic Field (mT)	110
Irradiation time per site (sec)	115
Total dose per site (J)	60
Total dose per muscle group (J)	180
Aperture of device (cm^2^)	33
Application mode	Cluster probe held stationary in skin contact with a 90-degree angle and slight pressure

#### Photobiomodulation therapy (PBMT) combined with static magnetic fields (sMF) (PBMT-sMF) – placebo

We performed the placebo treatments using the same device than active PBMT-sMF, employing the same application technique, irradiation sites and exposure (time). For placebo treatments, all the time-windows between treatments and the eccentric exercise protocol were employed, thus two volunteers were treated with placebo mode and 5 mins time-window, two were treated with placebo mode and 3 h time-window, three were treated with placebo mode and 6 h time-window, and three were treated with placebo mode and 1-day time-window.

#### Control group

In the control group the volunteers performed the exact same procedures performed by the volunteers of the other groups, but they did not receive any kind of treatment. We included this group in this study to ensure that the fact we have balanced different time-windows in placebo group would not represent a bias.

### Procedures

**Fig. 2:**
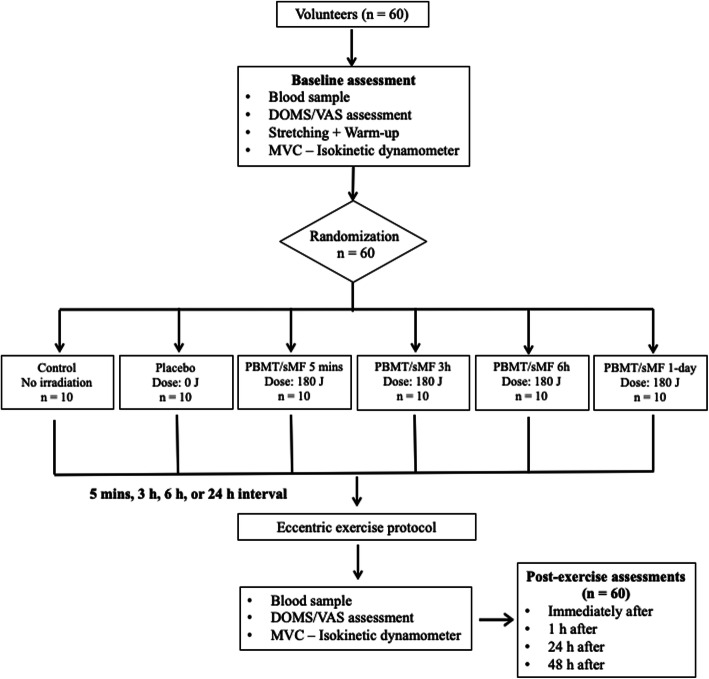
Procedures flowchart

The Consolidated Standards of Reporting Trials (CONSORT) flowchart that summarizes the experimental procedures and volunteers is shown in Fig. 2.

#### Blood samples collection

A qualified nurse collected blood samples (10 ml) from the antecubital vein of the volunteers to establish baseline values. We collected additional samples at immediately after (1 min), 1 h, 24 h, and 48 h after the eccentric contraction protocol. We centrifuged each sample at 3000 rpm for 20 min immediately after collection. We used pipettes to transfer the serum to Eppendorf® tubes, which were stored at − 80 °C until analysis. We utilized spectrophotometry and specific reagent kits (R&D Systems, Minneapolis, MN - USA) to analyze creatine kinase (CK) activity as an indirect marker of muscle damage.

#### Delayed onset muscle soreness (DOMS) intensity

We used as a self-rating assessment of volunteers DOMS intensity employing a visual analogue scale (VAS) of 100 mm. We obtained DOMS intensity at baseline, immediately after (1 min), 1 h, 24 h and 48 h after the eccentric exercise protocol.

#### Warm up

Prior to the maximum voluntary contraction (MVC) test, each volunteer performed a light warm up consisting in walking in a motorized treadmill for five minutes at 6 km/h, without inclination.

#### Peak torque obtained from maximum voluntary contraction (MVC)

We performed the MVC test on an isokinetic dynamometer to measure peak torque from maximum voluntary contraction and to evaluate muscle function immediately following the warm-up exercise. We positioned the volunteers seated upright on the isokinetic dynamometer (System 4, Biodex®, USA) with an angle of 100° between the trunk and hip and fixated by two straps crossing the trunk. We positioned the non-dominant leg of the volunteers with the knee at 60° of flexion (0° corresponds to complete knee extension) and fixated to the seat of the dynamometer by straps. We positioned the dominant leg of the volunteers at 100° of hip flexion and fixated as well. We also instructed the volunteers to cross their arms over the trunk and we positioned the axis of the dynamometer parallel to the center of the knee. The MVC test consisted of three five-second isometric contractions of the knee extensors of the non-dominant leg. We used the highest torque value of the three contractions (peak torque) for the statistical analysis. We firstly gave the instructions on how to execute the test, and the volunteers received verbal encouragement during the execution of the test. We repeated the MVC tests immediately after (1 min), 1 h, 24 h and 48 h after the eccentric exercise protocol. We carried out all MVC tests between 2 p.m. and 6 p.m.

#### Therapy application

We applied the therapy (both PBMT-sMF active or PBMT-sMF placebo) precisely in different time points after baseline MVC test to ensure that both MVC tests (in all time points tested) and eccentric exercise protocol would occur at the same hour of the day in all groups. Regarding the active PBMT-sMF, for the PBMT-sMF 5 mins group the irradiation was performed 24 h after baseline MVC, 5 min before the eccentric exercise protocol. For the PBMT-sMF 3 h group the irradiation was performed 21 h after baseline MVC, 3 h before the eccentric exercise protocol. For the PBMT-sMF 6 h group the irradiation was performed 18 h after baseline MVC, 6 h before the eccentric exercise protocol. And for the PBMT-sMF 1-day group the irradiation was performed immediately after baseline MVC, 24 h before the eccentric exercise protocol. Regarding the PBMT-sMF placebo the same procedures to ensure that MVC tests and eccentric exercise protocol would occur at the same hour of the day were followed. However, as mentioned before, two volunteers were treated with placebo mode and 5 mins time-window, two were treated with placebo mode and 3 h time-window, three were treated with placebo mode and 6 h time-window, and three were treated with placebo mode and 1-day time-window. We chose these time points trying to translate the findings of a previous animal study with mice [38].

#### Eccentric exercise protocol

We carried out the eccentric exercise protocol between 2 p.m. and 6 p.m. for all groups tested. Precisely 5 min after baseline MVC (Control group), or 5 min, 3 h, 6 h or 1-day (24 h) after PBMT-sMF irradiation (active or placebo), volunteers performed a single eccentric exercise protocol to induce skeletal muscle fatigue and exercise-induce muscle damage. The protocol consisted of 75 eccentric isokinetic contractions of the knee extensor musculature in the non-dominant leg (5 sets of 15 repetitions, 30-s rest interval between sets) at a velocity of 60°*sec^− 1^ in both the flexion and extension phases of the knee movements with a 60° range of motion (between 90° and 30° of knee flexion). At each contraction, the dynamometer automatically (passively) positioned the knee at 30°; the dynamometer then flexed the knee until reaching 90° [14–17, 34]. We instructed the volunteers to resist only against knee flexion movement imposed by the dynamometer with maximum force, and to do not perform any muscular contraction during the passive knee extension movement. We firstly gave instructions on how to execute the maneuver and the volunteers received verbal encouragement throughout the protocol. Despite the diversity of protocols proposed for the execution of eccentric exercises on isokinetic dynamometers, previous studies [14–17, 23, 34] employing PBMT and/or PBMT-sMF have utilized the same protocol that has demonstrated reliability and reproducibility for exercise-induced muscle damage. The researcher in charge was blinded to randomization and allocation of volunteers to the experimental groups.

### Outcomes

The primary outcome was the peak torque obtained from maximum voluntary contraction (MVC) and secondary outcomes were creatine kinase (CK) activity and delayed onset muscle soreness (DOMS) measured by VAS. We obtained both primary and secondary outcomes at baseline, immediately after (1 min), and 1 h, 24 h and 48 h after the eccentric exercise protocol. The assessors that collected all outcomes were not aware of volunteers’ allocation to their treatment groups.

### Characterization of sample

We calculated the sample size based on a previous study [15] that utilized the same kind of PBMT-sMF technology and that observed positive significant improvement in MVC, DOMS, and CK activity. For sample size calculation, we considered the β value of 20% and α of 5%. In study used as reference for sample size calculation [15], it was observed that PBMT-sMF led to increase maximum voluntary contraction - MVC (our primary outcome) to 336.9 N.m (± 27.9) post-exercise (Cohen’s *d* = 1.48), compared to baseline (286.6 ± 38.9). Thus, the calculation resulted in a sample of 10 volunteers per group, 60 volunteers in total.

### Statistical analysis

We conducted the statistical analysis following intention-to-treat principles [39]. The researcher that performed statistical analysis was blinded to randomization and allocation of volunteers to the experimental groups. We firstly tested data regarding normal distribution using Shapiro-Wilk test. Since the data showed normal distribution, for demographic data we used one-way ANOVA test (followed by Bonferroni post hoc test), and for primary and secondary outcomes, we performed a mixed design ANOVA (repeated design for time, non-repeated design for group) followed by Bonferroni post hoc test to test between group differences at each time point. We analyzed data in terms of both their absolute values and their percentage of change based on the values established in the baseline tests. The significance level was set at *p* < 0.05. In tables we expressed the data as mean and standard deviation (SD), and in graphs data are represented as mean and standard error of the mean (SEM).

## Results

### Anthropometrical and primary outcome baseline data

Sixty healthy, untrained, male subjects were recruited between May and July 2019 and completed all procedures. Among groups volunteers had a mean age ranging from 26.0 years old (±4.7) to 28.8 years old (±5.0), mean body weight ranging from 76.3 kg (±13.6) to 81.4 kg (15.7), mean height ranging from 175.2 cm (±5.1) to 177.2 cm (±6.2), and baseline MVC (primary outcome) ranging from 258.2 N.m (±22.3) to 282.1 N.m (±32.9). All participants received treatment according to the randomized allocation and were analyzed for all outcomes (*n* = 10 per group). The anthropometrical data of volunteers and primary outcome data at baseline were similar in all groups (*p* > 0.05) and are full described in Table 2.

**Table 2:** Anthropometrical and primary outcome baseline data (*n* = 60)

Variables	Control (n = 10)	Placebo (n = 10)	PBMT-sMF 5 mins (n = 10)	PBMT-sMF 3 h (n = 10)	PBMT-sMF 6 h (***n*** = 10)	PBMT-sMF 1-day (n = 10)	***P*** value
Age (Years)	26.7 (± 5.1)	27.7 (± 5.4)	27.3 (± 6.8)	28.8 (± 5.0)	27.0 (± 4.9)	26.0 (± 4.7)	0.9029
Body weight (kg)	77.4 (± 15.1)	80.3 (± 14.7)	78.6 (± 14.3)	81.4 (± 15.7)	76.3 (± 13.6)	79.1 (± 14.2)	0.9753
Body height (cm)	176.3 (± 6.5)	177.2 (± 6.2)	175.0 (± 7.6)	177.1 (± 5.5)	176.8 (± 8.0)	175.2 (± 5.1)	0.9566
MVC (N.m)	258.2 (± 22.3)	269.8 (± 25.7)	281.2 (± 19.1)	270.1 (± 22.4)	269.2 (± 22.2)	282.1 (± 32.9)	0.2748

Continuous variables are expressed as mean (SD). One-way ANOVA test and Bonferroni post hoc test

### Percentage of change in MVC

**Fig. 3:**
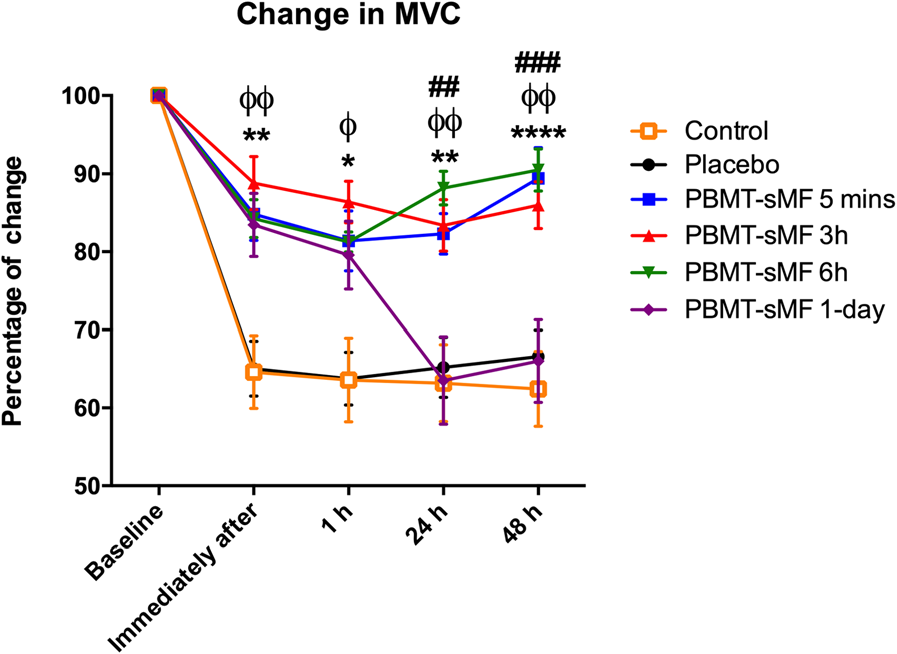
Percentage of change in MVC. Values are mean and error bars are SEM. ^*^ indicates significant difference compared to control (*p* < 0.05), ^**^ indicates significant difference compared to control (*p* < 0.01), ^****^ indicates significant difference compared to control (*p* < 0.0001); ^ϕ^ indicates significant difference compared to placebo (p < 0.05), ^ϕϕ^ indicates significant difference compared to placebo (p < 0.01); ^##^ indicates significant difference compared to PBMT-sMF 1-day (p < 0.01), ^###^ indicates significant difference compared to PBMT-sMF 1-day (*p* < 0.001). Mixed design ANOVA test with Bonferroni post hoc test (time = p < 0.0001, group = p < 0.0001, interaction = p < 0.0001)

All PBMT-sMF groups showed significantly increased percentage of change in MVC compared to both control and placebo group at immediately after (interaction *p* < 0.0001, pairwise comparisons *p* = 0.0025 to p < 0.0001) and 1 h after eccentric exercise (interaction p < 0.0001, pairwise comparisons *p* = 0.0180 to p < 0.0001). Besides that, PBMT-sMF 5 mins, 3 h and 6 h groups significantly increased MVC compared to control, placebo and PBMT-sMF 1-day groups at 24 h (interaction *p* < 0.0001, pairwise comparisons *p* = 0.0071 to p < 0.0001) and 48 h (interaction p < 0.0001, pairwise comparisons *p* = 0.0012 to p < 0.0001). Additionally, PBMT-sMF 1-day group was similar to the control and placebo groups at 24 h and 48 h (*p* > 0.05). Figure 3 summarized the data for percentage of change, and Table 3 summarized the data in absolute values and provides detailed *p* values for each comparison.

**Table 3:** Mean and standard deviation for primary and secondary outcomes in absolute values (n = 60)

Outcomes	Mean (± standard deviation)					
	Control (n = 10)	Placebo (n = 10)	PBMT-sMF 5 mins (***n*** = 10)	PBMT-sMF 3 h (n = 10)	PBMT-sMF 6 h (n = 10)	PBMT-sMF 1-day (n = 10)
**MVC**						
Baseline	258.2 (±22.3)	269.8 (±25.7)	281.2 (±19.1)	270.1 (±22.4)	269.2 (±22.2)	282.1 (±32.9)
Immediately after	166.8 (±24.5)	175.4 (±19.6)	239.6 (±41.6) ^aaaa, bbbb^	239.9 (±34.8) ^aaaa, bbbb^	226.7 (±28.1) ^aaa, bb^	234.0 (±35.9) ^aaaa, bbb^
1 h	164.2 (±28.0)	172.0 (±18.3)	230.1 (±44.6) ^aaaa, bbb^	233.5 (±32.0) ^aaaa, bbbb^	218.5 (±19.6) ^aaa, bb^	223.3 (±39.8) ^aaa, bb^
24 h	163.1 (±25.5)	175.9 (±21.4)	232.0 (±32.5) ^aaaa, bbb, ccc^	224.2 (±24.9) ^aaaa, bb, cc^	236.9 (±22.8) ^aaaa, bbbb, ccc^	176.6 (±42.2)
48 h	161.2 (±24.4)	179.6 (±19.2)	252.5 (±45.4) ^aaaa, bbbb, cccc^	231.2 (±22.9) ^aaaa, bb, cc^	242.2 (±14.3) ^aaaa, bbbb, ccc^	183.5 (±41.2)
**CK activity**						
Baseline	67.2 (±25.7)	65.7 (±23.2)	67.2 (±26.5)	50.9 (±29.9)	63.9 (±35.5)	68.4 (±32.2)
Immediately after	232.6 (±35.4)	275.7 (±35.8)	90.0 (±31.7) ^aaaa, bbbb^	67.6 (±31.4) ^aaaa, bbbb^	95.0 (±49.9) ^aaaa, bbbb^	90.0 (±44.2) ^aaaa, bbbb^
1 h	348.8 (±38.5)	391.5 (±47.7)	75.7 (±26.1) ^aaaa, bbbb^	54.9 (±26.8) ^aaaa, bbbb^	86.9 (±51.0) ^aaaa, bbbb^	65.5 (±26.0) ^aaaa, bbbb^
24 h	519.4 (±55.4)	455.0 (±80.3)	174.8 (±54.4) ^aaaa, bbbb, cccc^	128.0 (±25.5) ^aaaa, bbbb, cccc^	173.4 (±51.5) ^aaaa, bbbb, cccc^	298.7 (±55.7) ^aaaa, bbbb^
48 h	769.6 (±63.6)	823.6 (±91.5)	181.6 (±64.2) ^aaaa, bbbb, cccc^	138.1 (±96.6) ^aaaa, bbbb, cccc^	161.1 (±65.7) ^aaaa, bbbb, cccc^	693.3 (±72.4) ^a, bbbb^
**DOMS intensity**						
Baseline	0.0 (±0.0)	0.0 (±0.0)	0.0 (±0.0)	0.0 (±0.0)	0.0 (±0.0)	0.0 (±0.0)
Immediately after	51.3 (±12.9)	53.9 (±14.2)	15.0 (±9.6) ^aaaa, bbbb, cccc^	16.5 (±12.4) ^aaaa, bbbb, cccc^	18.5 (±10.0) ^aaaa, bbbb, cccc^	42.6 (±15.2)
1 h	60.5 (±12.4)	65.5 (±13.4)	12.4 (±11.0) ^aaaa, bbbb, cccc^	14.4 (±8.1) ^aaaa, bbbb, ccc^	13.1 (±9.5) ^aaaa, bbbb, ccc^	36.7 (±18.2) ^aaa, bbbb^
24 h	65.7 (±13.2)	68.4 (±13.3)	21.3 (±9.2) ^aaaa, bbbb, cccc^	16.2 (±7.4) ^aaaa, bbbb, cccc^	17.8 (±14.1) ^aaaa, bbbb, cccc^	46.6 (±23.1) ^aa, bbb^
48 h	71.4 (±14.2)	76.2 (±15.1)	21.3 (±9.3) ^aaaa, bbbb, cccc^	27.1 (±11.9) ^aaaa, bbbb, cccc^	20.5 (±13.5) ^aaaa, bbbb, cccc^	64.6 (±12.3)

Continuous variables are expressed as mean (±SD)

*MVC* Maximum Voluntary Contraction (time = p < 0.0001, group = p < 0.0001, interaction = p < 0.0001); *CK* Creatine Kinase (time = p < 0.0001, group = *p* < 0.0001, interaction = *p* < 0.0001); *DOMS* Delayed Onset Muscle Soreness (time = p < 0.0001, group = p < 0.0001, interaction = p < 0.0001)

^a^ p < 0.05 compared to control, ^aa^ p < 0.01 compared to control, ^aaa^ p < 0.001 compared to control, ^aaaa^ p < 0.0001 compared to control; ^bb^ *p* < 0.01 compared to placebo, ^bbb^ *p* < 0.001 compared to placebo, ^bbbb^ p < 0.0001 compared to placebo; ^cc^ p < 0.01 compared to PBMT-sMF 1-day, ^ccc^ p < 0.001 compared to PBMT-sMF 1-day, ^cccc^ p < 0.0001 compared to PBMT-sMF 1-day. Mixed design ANOVA test with Bonferroni post hoc test

### Percentage of change in CK

**Fig. 4:**
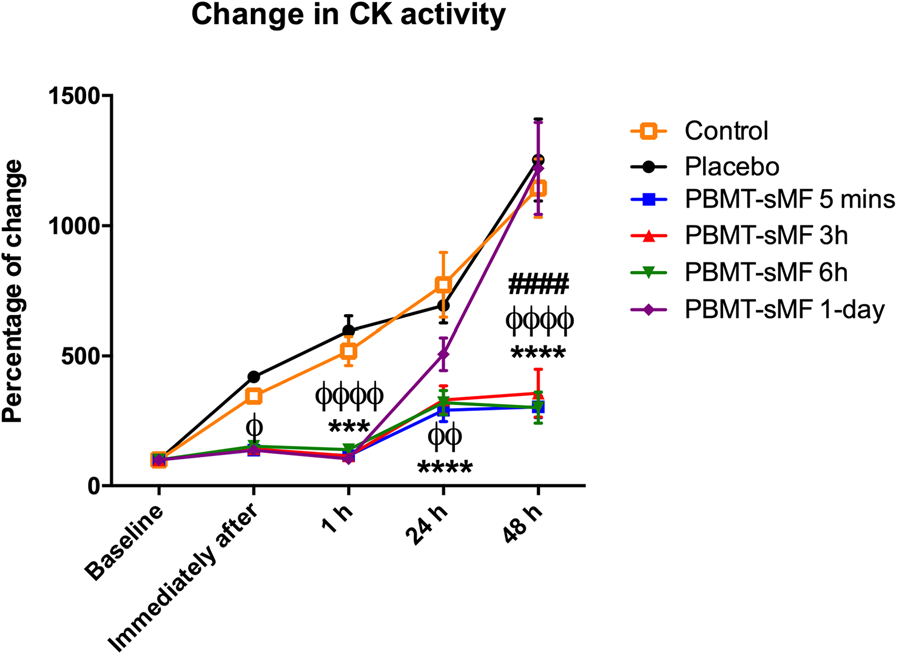
Percentage of change in CK activity. Values are mean and error bars are SEM. ^***^ indicates significant difference compared to control (p < 0.001), ^****^ indicates significant difference compared to control (p < 0.0001); ^ϕ^ indicates significant difference compared to placebo (p < 0.05), ^ϕϕ^ indicates significant difference compared to placebo (p < 0.01), ^ϕϕϕϕ^ indicates significant difference compared to placebo (p < 0.0001); ^####^ indicates significant difference compared to PBMT-sMF 1-day (p < 0.0001). Mixed design ANOVA test with Bonferroni post hoc test (time = p < 0.0001, group = p < 0.0001, interaction = p < 0.0001)

Significant differences were observed between all PBMT-sMF groups compared to placebo group for the percentage of change of CK activity immediately after the eccentric exercise protocol (interaction *p* < 0.0001, pairwise comparisons *p* = 0.0484 to *p* = 0.0285), however there were not observed differences compared to the control group (p > 0.05) at this timepoint. At 1 h after the eccentric exercise protocol all PBMT-sMF groups showed decreased change in CK activity compared to both control and placebo groups (interaction *p* < 0.0001, pairwise comparisons *p* = 0.0005 to p < 0.0001). PBMT-sMF 5 mins, 3 h and 6 h groups also showed decreased change in CK activity when compared to both control and placebo groups at 24 h after the eccentric exercise protocol (interaction *p* < 0.0001, pairwise comparisons *p* = 0.0011 to *p* < 0.0001). Moreover, PBMT-sMF 5 mins, 3 h and 6 h groups showed decreased change in CK activity when compared to control, placebo, and PBMT-sMF 1-day groups at 48 h (interaction *p* < 0.0001, pairwise comparisons p < 0.0001). PBMT-sMF 1-day group was similar to the control and placebo groups at 24 h and 48 h (*p* > 0.05). Figure 4 summarized the data for percentage of change, and Table 3 summarized the data in absolute values and provides detailed *p* values for each comparison.

### Data in absolute values

Mixed design ANOVA test showed statistically significant differences for MVC (time = p < 0.0001, group = p < 0.0001, interaction = p < 0.0001), CK (time = p < 0.0001, group = p < 0.0001, interaction = p < 0.0001) and DOMS (time = p < 0.0001, group = p < 0.0001, interaction = p < 0.0001). All the means, standard deviations, and between group comparisons for primary and secondary outcomes in absolute values are summarized in Table 3. Participants did not report any adverse events in this trial.

### DOMS

As can be observed in Table 3, all groups started the trial with no pain [40], and both control and placebo groups showed a progressive increase in DOMS over the time course of the study finishing the trial with moderate [40] and severe pain [40], respectively. PBMT-sMF 1-day also showed a progressive increase in DOMS over the time course of the study finishing the trial with moderate pain [40]. On the other hand, PBMT-sMF 5 mins, 3 h and 6 h groups did not show the same increase in DOMS, remaining over the whole post-exercise time course of the trial with mild pain [40].

## Discussion

To the best of our knowledge, this is the first study that investigates four time-response windows for the use of PBMT-sMF when used as an ergogenic agent before exercise, and also to investigate how long the effects last after performing the exercise. Our findings indicate that the PBMT-sMF device tested in this study led to similar outcomes for the 5 mins, 3 h and 6 h time-response windows. However, the 1-day time-response window was only able to increase MVC (primary outcome) up to 1 h after the eccentric exercise protocol. It suggests that PBMT-sMF effects start to decrease when the time between irradiation and exercise takes longer. Our outcomes for the PBMT-sMF 5 mins, 3 h and 6 h groups are very similar to the findings previously observed with a similar technology [15], where this same 180 J total dose led to increased MVC, decreased DOMS, and kept CK activity closer to baseline levels. However, in the previous study [15] PBMT-sMF was only used immediately before the eccentric exercise protocol.

It is important to highlight that the only one previous randomized controlled trial [35] that has similar aims than ours, only investigated two time-response windows (5 min and/or 6 h), used a different dose than ours (270 J to the quadriceps), used isolated PBMT, and only investigated muscular performance aspects through isokinetic dynamometer and muscle activation through electromyography during and immediately after the exercise protocol. Rossato et al. [35] observed that PBMT applied 6 h before plus immediately before a high-intensity exercise protocol was able to reduce the strength fall in healthy and physically active subjects. Our outcomes agreed that PBMT-sMF is able to increase performance, however, with a single irradiation applied at 5 mins, 3 h or 6 h before a high intensity exercise protocol with decreased effects when PBMT-sMF is applied 1-day (24 h) before the exercise activity. We believe that the extensive optimization of parameters previously performed with the same PBMT-sMF technology used in our study [10, 15, 19–21], and the increased effects of the synergistic use of three wavelengths [3], besides the combination of PBMT with sMF [28], which allowed for only one treatment within 6 h to have positive effects instead two treatments (6 h and 5 mins before) as previously observed [35]. Thus, we believe that our study brings new knowledge to the existing evidence body in literature.

In fact, the use of three wavelengths synergistically can lead to optimized outcomes in exercise performance enhancement, since different wavelengths have different time-response windows in intact (non-injured) skeletal muscles, as previously demonstrated in an in vivo study [3]. Therefore, as suggested previously [3] the simultaneous use of three wavelengths in different bands of spectrum (from red to near infrared) can enhance cytochrome c oxidase activity, and consequently mitochondrial function and ATP production, from 5 min to 24 h after irradiation, which partially explains our outcomes. Moreover, it has been demonstrated in cellular level that sMF amplifies the effect of PBMT, and that the synergistic effect of PBMT (with three wavelengths) and sMF leads to enhanced electron transfer and as consequence increased mitochondrial respiratory chain activity and increased ATP production are observed [28]. Other biological effects that can explain our findings are related to the direct release of nitric oxide from hemoglobin and nitrosylated myoglobin [4] or production by NO synthase [41] causing vasodilatation, increased blood flow, and increased oxygen availability [42].

Another important aspect to be discussed is regarding the direct translation of outcomes related to the kinetics of the effect of PBMT from in vitro and in vivo animal studies to the clinical practice. A previous study using myotubes formed from C2C12 mouse muscle cells (in vitro) and exposed to PBMT [43], at the same time-response windows tested in our study, suggested an optimum time-response window of 3–6 h in order to PBMT stimulate muscle cells aiming ergogenic effects. Similarly, the same research group [38] also tested in vivo (mice) the same time-response windows than we tested in our study. Authors did not observe effects at 5 mins time-response window, but found increased muscular ATP and increased performance in mice when 3 h, 6 h and 24 h (1-day) time-response windows were used, with better results in favor of 6 h time-response window. Interestingly, the clinical outcomes observed both by Rossato et al. [35] and by our findings did not corroborate with the outcomes observed by Ferraresi et al. both in cultured myotubes (in vitro) [43] and mice (in vivo) [38]. Despite Ferraresi et al. [38] state that “*Although the time response in mice and humans is not the same, athletes might consider applying LEDT at 6 h before competition*.”, the outcomes from both randomized controlled trials performed with human volunteers showed that indeed the time-response window in mice and humans is not exactly the same. In addition, the results from both randomized controlled trials also stress the point that clinical recommendations cannot be made based in findings from animal experiments nor cell culture studies. In fact, clinical recommendations shall be made based on high quality randomized controlled trials with adequate sample size and systematic reviews, preferably with meta-analysis.

Our study presents some limitations since the experimental design was limited to four time-response windows, the longest time-response window tested was 1-day (24 h), the tests were carried out in a controlled environment employing a standardized exercise protocol, and biochemical markers of oxidative stress or inflammation [44] were not investigated. Moreover, our study does not provide mechanistic insights behind the ergogenic effects of PBMT-sMF. On the other hand, as strengths of this study we can highlight that it is a randomized, placebo-controlled, and prospectively registered clinical trial, therefore it has high methodological quality. Furthermore, it has a triple blind design, and the sample size was calculated based on a previous study to provide the appropriate statistical power to detect precise differences for the primary outcome of the study. Moreover, the parameters were previously optimized and the dose used is in accordance with the current evidence [2, 33].

Finally, the findings of this manuscript elucidate the optimal time-response window for exercise performance when this device with three wavelengths and static magnetic fields is employed. Our outcomes also open several possibilities for the use of PBMT-sMF in different clinical settings related to sports and exercise, since different sports modalities have different dynamics before matches and competitions, such as warm-up, time expended in locker rooms, etc. We also think that the possibility to have until 6 h to use PBMT-sMF before a specific match or a competition and still have positive effects until 48 h after that is very helpful in making the use of PBMT-sMF feasible for athletic trainers, and physical therapists, which very often have only one device available to treat a large number of athletes. Therefore, the use of this device can be recommended mainly from 5 min up to 6 h before performing the exercise or sports activity, and it is important to highlight that some non-optimal positive results are still observed when the treatment is performed 24 h before the activity. Further research is warranted to confirm these findings in different sports-specific scenarios.

## Conclusions

PBMT-sMF with the combination of parameters tested in this study can be used from 5 min to 1-day (24 h) before exercise to promote ergogenic effects. However, the effects of PBMT-sMF start to decrease when a 1-day (24 h) time-response window is used. Our outcomes also showed that PBMT-sMF ergogenic effects can last up to 54 h (6 h before exercise + 48 h follow up = 54 h) after treatment.

## Data Availability

The datasets used and/or analyzed during the current study are available from the corresponding author on reasonable request.

## Abbreviations

PBMT: Photobiomodulation therapy
LLLT: Low-level laser therapy
LEDT: Light emitting diodes therapy
lasers: light amplification by stimulated emission of radiation
LEDs: Light-emitting diodes
sMF: static magnetic fields
PBMT-sMF: Photobiomodulation therapy combined with static magnetic fields
CK: Creatine kinase
DOMS: Delayed Onset Muscle Soreness
VAS: Visual analogue scale
MVC: Maximum voluntary contraction
